# Data on the kinetics of *in vitro* assembled chromatin

**DOI:** 10.1016/j.dib.2016.05.057

**Published:** 2016-06-01

**Authors:** Moritz Carl Völker-Albert, Miriam Caroline Pusch, Andreas Schmidt, Axel Imhof

**Affiliations:** BioMedical Center and Center for Integrated Protein Sciences Munich, Ludwig-Maximilians-University of Munich, Großhaderner Straße 9, 82152 Planegg-Martinsried, Germany

**Keywords:** chromatin assembly, quantitative proteomics, in-vitro, Drosophila melanogaster

## Abstract

Here, we use LC–MS/MS and SWATH-MS to describe the kinetics of *in vitro* assembled chromatin supported by an embryo extract prepared from preblastoderm *Drosophila melanogaster* embryos (DREX). This system allows easy manipulation of distinct aspects of chromatin assembly such as post-translational histone modifications, the levels of histone chaperones and the concentration of distinct DNA binding factors. In total, 480 proteins have been quantified as chromatin enriched factors and their binding kinetics have been monitored in the time course of 15 min, 1 h and 4 h of chromatin assembly.

The data accompanying the manuscript on this approach, Völker-Albert et al., 2016 “*A quantitative proteomic analysis of in vitro assembled chromatin*” [1], has been deposited to the ProteomeXchange Consortium (http://www.proteomexchange.org) via the PRIDE partner repository with the dataset identifier submission number PRIDE: PXD002537 and PRIDE: PXD003445.

**Specifications Table**

**Table:** 

Subject area	*Biology*
More specific subject area	*Epigenetics and chromatin organisation*
Type of data	*Figure*
How data was acquired	*Mass spectrometry. LC–MS/MS and SWATH-MS (LC: Ultimate 3000 HPLC system, Thermo-Fisher Scientific. MS: LTQ Orbitrap XL, Thermo-Fisher Scientific, TripleTOF 6600, Sciex)*
Data format	Raw (*.raw, *.wiff, *wiff.scan), Annotated (*.txt, *.zip, *.mgf)
Experimental factors	*in vitro chromatin assembly for the duration of 15 min, 1 h, 4 h and subsequent proteomic quantitative analysis*
Experimental features	*in vitro chromatin assembly, micrococcal nuclease eluted chromatin from streptavidin beads, tryptic in solution digestion, LC–MS/MS or SWATH-MS of peptides, quantitative analysis of chromatin enriched factors compared to beads-only control with Perseus software*
Data source location	*Munich, Germany*
Data accessibility	Data is within this article and uploaded to the ProteomeXchange Consortium webpage. Dataset identifier: PRIDE: PXD002537 and PRIDE: PXD003445.

## Value of the data

• Comprehensive dataset of label-free quantitative proteomics with a well-described *in vitro* chromatin assembly system from *Drosophila melanogaster containing SWATH libraries for follow-up analysis*
• By choosing 3 different time points, data are able to describe the kinetics of the chromatin assembly *in vitro* at an unprecedented depth
• The use of a quantitative method to investigate chromatin assembly *in vitro* resembles many aspects of replication dependent chromatin synthesis *in vivo* and can therefore be used to dissect key regulatory steps in chromatin assembly which are often difficult to investigate in living cells.

## 1 Data

**Fig. 1:**
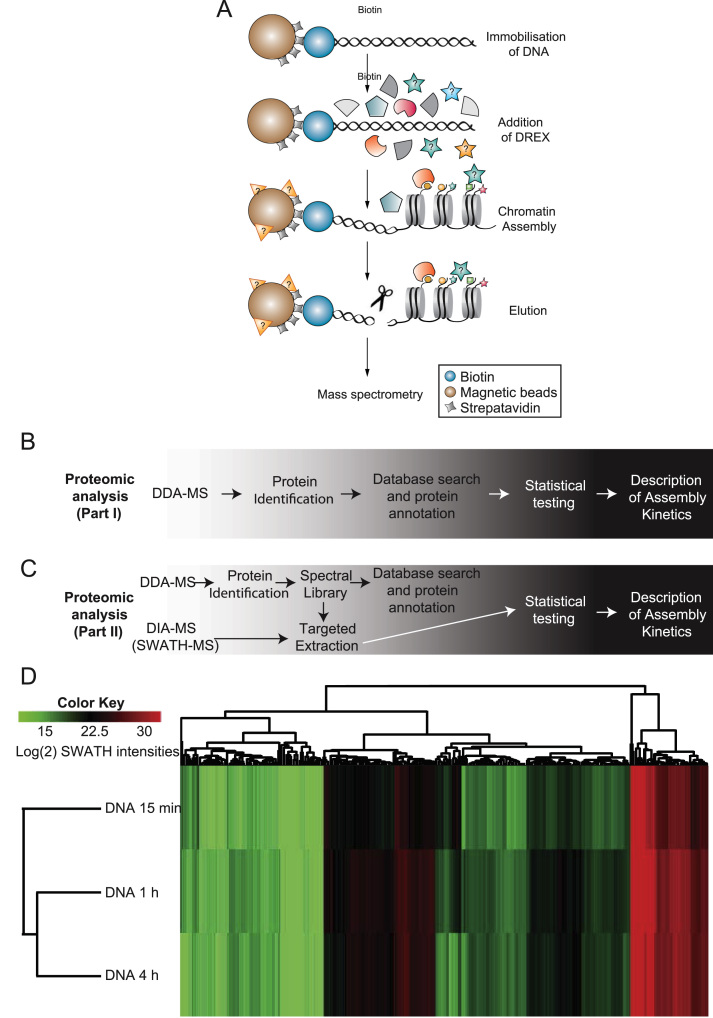
(A) Workflow of DREX-mediated chromatin assembly. Linearized and biotinylated DNA is immobilized on streptavidin-coated, paramagnetic beads. It is incubated with Drosophila embryo extract (DREX). The assembly is stopped in regularly time intervals to follow protein binding kinetics. (B) Data-dependent acquisition of chromatin assembly. Workflow of proteomic analysis for DDA-MS mode. (C) Data-dependent acquisition of chromatin assembly for library generation and quantitation with data-independent acquisition. Workflow of proteomic analysis for DIA-MS mode (SWATH-MS). (D) The Kinetics of Chromatin Assembly. Heatmap illustrating Log 2(*x*) transformed median averaged SWATH intensities from three replicates after Euclidean clustering. Columns indicate proteins, rows represent different conditions of chromatin assembly. Red and green colors indicate SWATH intensities.

DNA has been assembled into chromatin (Fig. 1A) for 15 min, 1 h and 4 h within three biological replicates by means of three independently collected Drosophila extracts (DREX) http://proteomecentral.proteomexchange.org/cgi/GetDataset?ID=PXD002537, http://proteomecentral.proteomexchange.org/cgi/GetDataset?ID=PXD003445.

Assembled chromatin was either analyzed with DDA-MS on a LTQ Orbitrap XL (Fig. 1B) or it was analyzed with DIA-MS on a TTOF 6600 (Fig. 1C). Annotated spectra can be viewed at MS-Viewer (http://prospector2.ucsf.edu/prospector/cgi-bin/msform.cgi?form=msviewer). Spectra from DREX samples can be accessed with search key: hlyzvamoxh. Spectra from chromatin samples can be accessed with search key: 3keubvytc7

An ion library generated from DIA-MS runs from three DREX assembly extracts and the proteins bound to chromatin after 4 h assembly is present in both public available data sets. By means of DIA-MS in combination with a conservative statistical analysis, we were able to quantify 480 chromatin-enriched proteins compared to the negative control [1] (Fig. 1 D).

## 2 Experimental design, materials and methods

### 2.1 Preparation of Drosophila embryonic extract [DREX]

*Drosophila melanogaster* embryos were collected on agar trays with yeast paste 0–90 min after egg-laying. Using a brush and sieves with descending mesh size (0.71 mm, 0.355 mm, 0.125 mm), embryos were rinsed with cold tap water and allowed to settle into ice-cold embryo wash buffer (0.7% NaCl, 0.05% Triton X-100) to arrest further development. After five successive collections, the wash buffer was decanted and replaced with wash buffer at room temperature. For dechorionation of the embryos, the volume was adjusted to 200 ml and 60 ml of 13% hypochlorite solution was added. The embryos were stirred vigorously for 4 min on a magnetic stirrer, poured back into the collection sieve (0.125 mm), and rinsed with tap water for 5 min. Embryos were allowed to settle in 200 ml of wash buffer for about 3 min. Afterwards the supernatant containing the chorions was removed. Following two more settlings in 0.7% NaCl and in extract buffer (10 mM HEPES [pH 7.6], 10 mM KCl, 1.5 mM MgCl2, 0.5 mM EGTA, 10% glycerol, 10 mM 3-glycero-phosphate, 1 mM dithiothreitol [DTT], and 0.2 mM phenylmethylsulfonyl fluoride [PMSF], added freshly) at 4 °C, the embryos were settled in extract buffer in a 60 ml glass homogenizer on ice. The volume of the packed embryos was estimated before the supernatant was aspirated, leaving packed embryos and additional 2 ml buffer on top. Homogenization was performed with one stroke at 3000 rpm and 10 strokes at 1500 rpm with a pestle connected to a drill press. The homogenate was supplemented with MgCl_2_ to a final MgCl_2_ concentration of 5 mM. Nuclei were pelleted by centrifugation for 10 min at 10,000 rpm in a SS34 rotor. (Sorvall, Thermo-Fisher Scientific, Waltham, USA). The supernatant was centrifuged again for 2 h at 45,000 rpm in a chilled SW 56 rotor (Beckman-Coulter, Germany). The clear extract was isolated with a syringe, avoiding the top layer of lipids. Extract aliquots were frozen in liquid nitrogen. Protein concentration was determined by Nanodrop measurement and titration with chromatin assembly experiments.

### 2.2 Biotinylation of DNA

To obtain linearized and biotinylated DNA, we used a plasmid DNA that contains oligomers of the sea urchin 5S rDNA positioning sequence. 500 µg plasmid DNA were linearized using the restriction enzyme SacI. Completion of the digest was analyzed by agarose gel electrophoresis. Upon completion of the plasmid digestion, we added the restriction enzyme XbaI to the reaction and incubated for at least 3 h at 37 °C. Subsequently, the DNA was precipitated and purified, followed by incubation with 80 mM dCTP and dGTP, 3 mM biotinylated dUTP and dATP and the Klenow Polymerase. To purify DNA from excessive nucleotides and enzyme, we used G50 Sepharose colums (Roche) according to the manufacturers protocol. Finally, DNA concentration was measured and adjusted to 200 ng/µl.

### 2.3 Chromatin assembly on immobilized DNA

4 μg DNA was immobilized onto 120 µl M280 paramagnetic streptavidin beads (Invitrogen) in EX100 buffer (10 mM HEPES [pH 7.6], 100 mM NaCl, 1.5 mM MgCl2, 0.5 mM EGTA, 10% [vol/vol] glycerol, 0.2 mM PMSF, 1 mM DTT) for 1 h. Beads were extensively washed and blocked with BSA for 30 min (1.75 g/l) in EX100. After another washing step in EX-NP40 (10 mM Hepes pH 7.6, 1.5 mM MgCl_2_, 0.5 mM EGTA, 10% (v/v) glycerol, 0.05% NP-40) beads were resuspended in a total volume of 240 μl containing 45 μl DREX and an ATP regenerating system (3 mM ATP, 30 mM creatine phosphate, 10 μg creatine kinase/ml, 3 mM MgCl2, and 1 mM DTT). For time-resolved studies, the assembly reaction was incubated at 26 °C for 15 min, 1 h and 4 h, respectively. After two stringent wash steps with EX200, beads were resuspended in elution buffer (EX100 with 0.5 U/μl MNase and 2 mM CaCl_2_). The supernatant after 10 min of MNase-mediated elution was subjected to mass spectrometry-based protein quantitation.

### 2.4 Preparation of MS samples for proteomics analysis

Assembled chromatin was subjected to mass spectrometry analysis. 90% of the chromatin bound proteins were subjected to MS-sample preparation. Proteins were denatured in 3 M Urea, 1 M Thiourea and 25 mM DTT for 2 h at 20 °C followed by an incubation for 30 min in a dark place with a final concentration of 25 mM iodoacetamide at 20 °C to carbamidomethylate sulfhydryl groups of free cysteine. Subsequently, DTT was added to a final concentration of 50 mM and incubated for 30 min at 20 °C. The samples were diluted with 100 mM ammonium bicarbonate to lower the urea concentration below 1 M for tryptic cleavage with 200 ng of trypsin (Promega) in 50 mM ammonium bicarbonate. Digestion was completed after 14 h at 25 °C. 10% of the tryptic peptide mixture were acidified using trifluoroacetic acid (TFA) and desalted using C18 stage tips prior to mass spectrometry analyses and redissolved in 0.2% TFA [2]. The resulting liquid, containing the digested peptides, was dried and redissolved in 17 μl of 0.2% TFA and stored at –20 °C until further processing.

### 2.5 Proteomic analysis via LC–MS/MS on Orbitrap mass spectrometer

The peptide mixture resulting from tryptic cleavage was injected onto an Ultimate 3000 HPLC system equipped with a C18 trapping column (C18 PepMap, 5 mm×0.3 mm×5 μm, 100 Å) and an analytical column (C18RP Reposil-Pur AQ, 120 mm×0.075 mm×2.4 μm, 120 Å, Dr. Maisch, Germany) packed into an ESI-emitter tip (New Objective, USA). First, the peptide mixture was desalted on the trapping column for 7 min at a flow rate of 25 μl/min (0.1% FA). For peptide separation a linear gradient from 5% to 40% B (HPLC solvents A: 0.1% FA, B: 80% ACN, 0.1% FA) was applied over a time of 120 min. The HPLC was online coupled to an LTQ Orbitrap XL mass spectrometer (Thermo-Fisher Scientific, USA).

The mass spectrometer was operated in DDA-mode employing a duty cycle of one survey scan in the orbitrap at 60,000 resolution followed by up to 6 tandem MS scans in the ion trap. Precursors were selected when they had a minimal intensity of 10,000 counts and a charge state of 2+ or higher. Previously analyzed precursors were excluded for 20 s within a mass window of −1.5 to +3.5 Da.

### 2.6 Proteomic analysis via LC–MS/MS on Q-TOF mass spectrometer

Samples were injected into an Ultimate 3000 HPLC system (Thermo Fisher Scientific) for nano-reversed phase separation of tryptic peptide mixtures before MS analysis. Peptides were desalted on a trapping column (5×0.3 mm^2^ inner diameter; packed with C18 PepMap100, 5 μm particle size, 100 Å pore diameter, Thermo-Fisher Scientific). The loading pump flow of 0.1% formic acid (FA) was set to 25 μl/min with a washing time of 10 min under isocratic conditions. Samples were separated on an analytical column (150×0.075 mm^2^ inner diameter; packed with C18RP Reposil-Pur AQ, 2.4 μm particle size, 100 Å pore diameter, Dr. Maisch) using a linear gradient from 4% to 40% B in 170 min with a gradient flow of 270 nl/minute. Solvents for sample separation were A: 0.1% FA in water and B: 80% ACN, 0.1% FA in water. The HPLC was directly coupled to the 6600 TripleTOF mass spectrometer using a nano-ESI source (both Sciex). A data-dependent method was selected for MS detection and fragmentation of eluting peptides comprising one survey scan for 225 ms from 300 to 1800 *m*/*z* and up to 40 tandem MS scans for putative precursors (100–1800 *m*/*z*). Precursors were selected according to their intensity. Previously fragmented precursors were excluded from reanalysis for 30 s.

### 2.7 Data analysis of data-dependent LC–MS experiments

DDA-MS data recorded on the LTQ Orbitrap mass spectrometer were processed with MaxQuant (version 1.2.2.5) using standard settings with the additional options LFQ and iBAQ (log fit) selected. Data were searched against a combined forward/reversed database (special amino acids: KR) including common contaminants for false-discovery rate filtering of peptide and protein identifications (Dmel_all translation r5.57, 30305 entries). The mass deviation for the precursor mass was set 20 ppm; fragment ions were matched within 0.5 Da mass accuracy. Fixed modifications of cysteine (Carbamidomethyl (C)) were included as well as variable modifications by oxidation of methionine and acetylation (Acetyl (Protein N-term); Oxidation (M)). Matches were filtered setting false peptide and protein (PSM FDR and protein FDR) hits to 1%. The minimum peptide length was allowed to be 6 amino acids, the minimum score for modified peptides was set to 40. For protein identification, one non-unique razor peptide was required, whereas protein quantitation was only performed if at least 2 razor peptides were associated with the protein hit. Prior to statistical analysis in Perseus, protein hits associated with the reversed database or common contaminants were filtered in the protein.groups.txt file.

Data-dependent experiments performed on the Q-TOF mass spectrometer were analyzed in MaxQuant (version 1.5.1.2) using the Andromeda search engine and the same flybase database as for Orbitrap data. The settings for database search were as follows: fixed modification carbamidomethyl (C), variable modification oxidation (M) and acetyl (protein N-term); Δmass=20 ppm for precursors, Δmass=50 ppm for TOF fragment ions. Peptide hits required a minimum length of 7 amino acids and a minimum score of 20 for unmodified and 40 for modified peptides. Resulting protein hits were FDR filtered for 1% false discoveries on the PSM level and up to 5% false protein hits. Settings for protein identification and quantitation were identical as for orbitrap data (see above).

### 2.8 SWATH data acquisition

Peptides from tryptic digestion were resuspended in 10 µl 0.1% TFA and injected into an Ultimate 3000 nano-chromatography system equipped with trapping column (C18 AcclaimPepMap, 5×0.2 mm^2^, 5 μm 100 Å) and a separation column (C18RP Reposil-Pur AQ, 150×0.075 mm×2.4 μm, 100 Å, Dr. Maisch, Germany) poured into a nano-ESI emitter tip (New Objective, Woburn MA). After washing for 10 min on the precolumn with 0.05% TFA, peptides were separated by a linear gradient from 4% to 40% B (solvent A 0.1% FA in water, solvent B 80% ACN, 0.1% FA in water) for 150 min at a flow rate of 270 nl/min. Eluting peptides were detected on a 6600 Triple TOF quadrupol-TOF hybrid mass spectrometer (Sciex, Framingham, MA). First, a mixture of all conditions was run in data-dependent mode to generate an ion library for the data-independent SWATH measurements and optimize the isolation window distribution over the mass range for SWATH-data acquisition. Data-dependent acquisition consisted of a survey scan and up to 40 tandem MS scans for precursors with charge 2–5 and more than 200 cps abundance. Rolling collision energy was set to generate peptide fragments. The overall cycle time for the DDA experiment was 2.676 s. Previously analyzed precursors were excluded from repeated fragmentation for 30 s employing a mass window of 20 ppm around the precursor mass.

MS data with data-independent SWATH acquisition were generated using the same HPLC conditions as used for the generation of the ion library. Based on the distribution of the *m*/*z* values of identified peptides in the ion library, the mass range from 300 to 1200 *m*/*z* was split into 40 SWATH mass windows to optimize the number of precursor ions per window. First, precursors were monitored from 300 to 1500 *m*/*z* in a survey scan of 50 ms, followed by the SWATH data acquisition for 65 ms/mass window, resulting in an overall cycle time of 2.7 s. The fragmentation energy was adjusted to fragment 2+ charged ions in the center of the mass window and a collision energy spread over 7 units was allowed. For data analysis, SWATH data were mapped to a protein database containing RT, peptide precursor and fragment ion information, that was generated in ProteinPilot 4.5 [Sciex] against the previously described drosophila database using database search settings described for MaxQuant 1.5.1.2. Settings for SWATH peak extraction in the Peak View 2.1 software [Sciex] were 5 peptides/protein with at least 6 mapping fragment ion signals, a confidence interval of 99% and FDR rate of 2%.

### 2.9 Statistical methods

Data were handled with Perseus software. Three biological replicates acquired with DDA-MS were analyzed for chromatin and DREX. All three biological replicates from 1 h and 4 h assembled chromatin or wtDREX were log 2(*x*) transformed. Missing values were replaced by random numbers from a standard deviation (width 0.3, shift −1.8).

DIA-MS SWATH intensities were normalized to the total area sum within the MarkerView software (version 1.2.1.1). In order to compare different assembly times, the median of three biological replicates was calculated for each protein and each time point.

### 2.10 Experimental design and statistical rationale

Chromatin assembly experiments have been performed in three biological replicates with three independently collected DREX. As negative controls, beads-only were incubated in three biological replicates with DREX. A pilot study in our lab revealed that three biological replicates enable us for a precise and statistical valid conclusion between chromatin assembly experiments and the composition of proteins during different time points of assembly. Based on biological function of the identified proteins, we altered the initial settings for statistical analysis to be (*s*(0)=3 and FDR=0.5%).
